# Avogadro: an advanced semantic chemical editor, visualization, and analysis platform

**DOI:** 10.1186/1758-2946-4-17

**Published:** 2012-08-13

**Authors:** Marcus D Hanwell, Donald E Curtis, David C Lonie, Tim Vandermeersch, Eva Zurek, Geoffrey R Hutchison

**Affiliations:** 1 Department of Chemistry, University of Pittsburgh, 219 Parkman Avenue, Pittsburgh, PA, 15260, USA; 2 Department of Scientific Computing, Kitware, Inc., 28 Corporate Drive, Clifton Park, NY, 12065, USA; 3 Department of Computer Science, Coe College, 1220 First Avenue NE, Cedar Rapids, Iowa 52402, USA; 4 Department of Chemistry, State University of New York at Buffalo, Buffalo, New York 14260-3000, USA; 5, Avogadro development team

## Abstract

**Background:**

The Avogadro project has developed an advanced molecule editor and visualizer designed for cross-platform use in computational chemistry, molecular modeling, bioinformatics, materials science, and related areas. It offers flexible, high quality rendering, and a powerful plugin architecture. Typical uses include building molecular structures, formatting input files, and analyzing output of a wide variety of computational chemistry packages. By using the CML file format as its native document type, Avogadro seeks to enhance the semantic accessibility of chemical data types.

**Results:**

The work presented here details the Avogadro library, which is a framework providing a code library and application programming interface (API) with three-dimensional visualization capabilities; and has direct applications to research and education in the fields of chemistry, physics, materials science, and biology. The Avogadro application provides a rich graphical interface using dynamically loaded plugins through the library itself. The application and library can each be extended by implementing a plugin module in C++ or Python to explore different visualization techniques, build/manipulate molecular structures, and interact with other programs. We describe some example extensions, one which uses a genetic algorithm to find stable crystal structures, and one which interfaces with the PackMol program to create packed, solvated structures for molecular dynamics simulations. The 1.0 release series of Avogadro is the main focus of the results discussed here.

**Conclusions:**

Avogadro offers a semantic chemical builder and platform for visualization and analysis. For users, it offers an easy-to-use builder, integrated support for downloading from common databases such as PubChem and the Protein Data Bank, extracting chemical data from a wide variety of formats, including computational chemistry output, and native, semantic support for the CML file format. For developers, it can be easily extended via a powerful plugin mechanism to support new features in organic chemistry, inorganic complexes, drug design, materials, biomolecules, and simulations. Avogadro is freely available under an open-source license from http://avogadro.openmolecules.net.

## Background

Many fields such as chemistry, materials science, physics, and biology, need efficient computer programs to both build and visualize molecular structures. The field of molecular graphics is dominated by viewers with little or no editing capabilities, such as RasMol [1], Jmol [2], PyMOL [3], VMD [4], QuteMol [5], BALLView [6], VESTA [7], and XCrySDen [8,9], among many others. The aforementioned viewers are all freely available, and most of them are available under open-source licenses and work on the most common operating systems (Linux, Apple Mac OS X, Microsoft Windows, and BSD).

The choice of software capable of building chemical structures in three dimensions is far smaller. There are existing commercial packages, such as CAChe/Scigress [10], ChemBio3D [11], GaussView [12], HyperChem [13], CrystalMaker [14], Materials Studio [15], and Spartan [16], which are polished and capable of constructing many different types of molecular structures. They are, however, not available for all operating systems (most of them only run on Microsoft Windows), and are not easily extensible, customized, or integrated into automated workflows. Licensing costs can be prohibitive. If the company were to change its direction or focus, this can lead to a loss of a significant research investment in a commercial product. Furthermore, in most cases, these programs use custom, proprietary file formats, and semantic and chemical data can be lost in conversion to other data formats.

The selection of free, open-source, cross-platform, three-dimensional, molecular builders was quite limited when the Avogadro project was founded in late 2006. Ghemical [17] was one of the only projects satisfying these needs at the time. Two of the authors (Hutchison and Curtis) contributed to Ghemical previously, but had found that it was not easily extensible. This led them to found a new project to address the issues they had observed in Ghemical and other packages. The Molden [18] application was also available, able to build up small molecules and analyze output from several quantum codes. However, it suffers from a restrictive license and it uses an antiquated graphical toolkit, which is not native on most modern operating systems.

Broad goals for the design of a molecular editor were identified following a case study of the available applications. One of the main issues with both commercial and open-source applications is a lack of extensibility; many of the applications also only work on one or two operating systems. The creation of an open and extensible framework that implements many of the necessary foundations for a molecular builder and visualizer would facilitate more effective research in this area. Further, the open, standardized Chemical Markup Language (CML) file format [19,20] would be used, to secure semantic and chemical data and allow easy interoperability with other chemistry software.

At the time of writing, it is apparent that other researchers have perceived similar needs. Several new applications are available today that focus on both building and visualizing molecular structure. These include CCP1GUI [21], Gabedit [22] and some highly specific editors such as MacMolPlt [23] which focus on particular computational packages (i.e., GAMESS-US for MacMolPlt). Whilst offering many interesting and useful features, these projects suffer from the same issues centering around effective reuse of existing code, well commented and documented code, and easy extension to add new features and adapt for specialized areas.

## Implementation

The Avogadro project was started in earnest in 2007, and over the first 5 years of development has been downloaded over 270,000 times [24], been translated into over 20 languages [25], and has over 20 contributors [26]. So far, it has been cited over 100 times [27], including applications in spectroscopy, catalysis, materials chemistry, theoretical chemistry, biochemistry, and molecular dynamics, among many others [28-47].

From the beginning, the project has strived to make a robust, flexible framework for both building and visualizing molecular structures. Much of the initial focus has been placed on preparing input and analyzing output from quantum calculations. Other applications such as preparing input for MD simulations and visualizing periodic structures will also be presented, demonstrating the flexibility of the Avogadro platform. The development team has also been members of the Blue Obelisk movement, following the three pillars outlined by the group: Open Data, Open Standards, and Open Source [48,49].

### Software architecture

One area that seems to suffer in many code bases in chemistry is software architecture. This can lead to less maintainable code, poor code reuse, and a much higher barrier to entry. Problems were identified in other projects with a view to minimize their impact when developing Avogadro. Modern software design processes were used in the initial planning stages of Avogadro, along with the choice of modern programming languages and libraries.

Avogadro has close ties to several other free, cross-platform, open-source projects to reuse as much code as is practical. These projects include Qt [50] to provide a free, cross-platform graphical toolkit; Open Babel [51] for chemical file input/output, geometry optimization, and other chemical perception; Eigen [52] for matrix and vector mathematics; OpenGL/GLSL for real-time, three-dimensional rendering; and POV-Ray for ray-traced rendering.

Based on the previous experience of the authors and a review of available programs at the time, several fundamental choices were made. The C++ programming language; the Qt graphical toolkit; OpenGL for 3D visualization; CMake as the build system; and Open Babel as the chemical library. Using this combination of languages and libraries requires the project to be licensed under the GNU GPLv2 [53] license and made openly available to all.

The core of Avogadro is written in portable C++ code with platform-specific differences abstracted away by Qt, OpenGL, and Open Babel. The CMake build system makes the build process relatively simple on all supported platforms. Avogadro has been successfully built and tested on Linux, Apple Mac OS X, and Microsoft Windows in common 32 and 64 bit hardware architectures.

The Avogadro framework uses the model, view, controller paradigm. The model is comprised of the core data classes such as Molecule, Atom, and Bond, views are made up of the engine/display plugins, and controllers are the tools (interactive mouse) and extensions (non-interactive, form based/menu based). Every plugin has full access to the core data model, but view and controller plugins are conceptually different; views are responsible for displaying data and controllers are responsible for modifying/changing data.

Plugins rely on Avogadro’s set of programming interfaces and almost all functionality is implemented in self contained plugins that are loaded at runtime. The majority of plugins distributed with Avogadro are written in C++, but the API is also available in the Python scripting language. This allows for a great deal of choice in how plugins are implemented. Each plugin is a singleton class that implements a particular set of functions–depending on the type of plugin–which allows for features to be implemented in a very modular way.

Over the last few years Avogadro development has started to use nightly builds of the latest version of the code in order to automatically flag issues introduced in new commits. Code review was also introduced in order to add a review step before new code is merged, along with softening the line between someone with commit rights and someone without (anyone can propose and upload a patch, but a small group can choose if/when the patch will be merged). Some automated testing has been added, but coverage at this point remains relatively low. API documentation is automatically generated from comments in the code using Doxygen.

### Plugin interface

**Figure 1:**
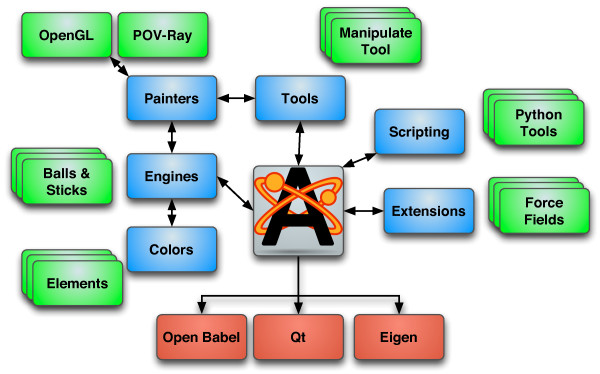
**General code architecture of Avogadro.** General code architecture of Avogadro, indicating major plugin interfaces for colors, display engines, tools, and extensions. Red boxes indicate code dependencies of Avogadro, blue boxes indicate plugin API classes, and green boxes inidicate examples of each plugin type.

Avogadro plugins are divided into four different types corresponding to four main classes that derive from this common base class, specializing their interface for specific activities (Figure 1). The Avogadro::Color base class defines the virtual interface for applying colors to atoms, bonds, and other properties. Avogadro::Engine defines the common interface for all display types in Avogadro: simple ball and stick, Van der Waals visualizations, surfaces, and force visualizations. The Avogadro::Tool base class provides the interface for all interactive tools, focusing principally on mouse and keyboard interaction with Avogadro. Examples of tool plugins include the draw tool used to draw molecules atom by atom, and the navigation tool used to pan, rotate, and scale the view of the molecule. There are also several specialized tools such as the alignment tool.

Finally there is the Avogadro::Extension class, which defines the interface for dialog based plugins. These extensions can interact with the molecule, and are used for a variety of purposes from molecule properties dialogs to input file generation dialogs for many quantum codes including NWChem, Gaussian, GAMESS, and others. This class of plugin is also applied to file import, and network aware extensions querying web databases for structures given their common name for example.

At start up, several standard directories, which may be customized, are searched for plugins. The Qt plugin framework is used to check that the plugins have a recent enough version to be loaded, and the plugin type can be deduced once loaded. The user interface is then populated with appropriate entries; tools are added to the main toolbar using their embedded icons, display types are added to the display type list, and menu entries are added for all loaded extensions.

The tool and display type plugins can both (optionally) provide a dialog for configuring the plugin. Dialogs are specific to each plugin and integrated into the user interface.

### Display types

Display plugins are referred to as “engines” internally. Their primary focus is rendering graphics to the screen. As is the case with most molecular graphics, a large portion of the geometric primitives are spheres and cylinders, typically used to represent atoms and bonds. There are many other properties that can be rendered using the display type plugins, for example, some of the engines also convey information about the underlying data the geometric primitives represent to allow for the molecule to be edited. Table 1 shows a summary of the display plugins distributed with Avogadro.

**Table 1:** List of default display type (engine) plugins

**Name**	**Description**
Axes	Renders x, y, z Cartesian axes from the origin
Ball and Stick	Standard ball and stick representation
Cartoon	Secondary biological structure (*α*helix and *β*sheet)
Dipole	Render direction/magnitude of dipole
	moment if present
Force	Renders arrows showing forces on atoms from
	force field
Hydrogen Bond	Renders hydrogen bonds as dotted lines
Label	Shows labels on atoms and bonds, configurable
Overlay	Overlay of color gradient used for electrostatic
	properties
Polygon	Renders closed polygons of metallic centers
Ribbon	Basic secondary structure ribbon rendering
Ring	Renders rings in structure, different colors
	depending on ring size
Simple Wireframe	Very simple wireframe display
Sticks	Stick or liquorice rendering style for atoms
	and bonds
Surface	Renders triangular isosurface meshes
Van der Waals	Van der Waals sphere rendering (no bonds,
Spheres	space-filling)
Wireframe	Wireframe with more features such as bond order
	rendering

**Figure 2:**
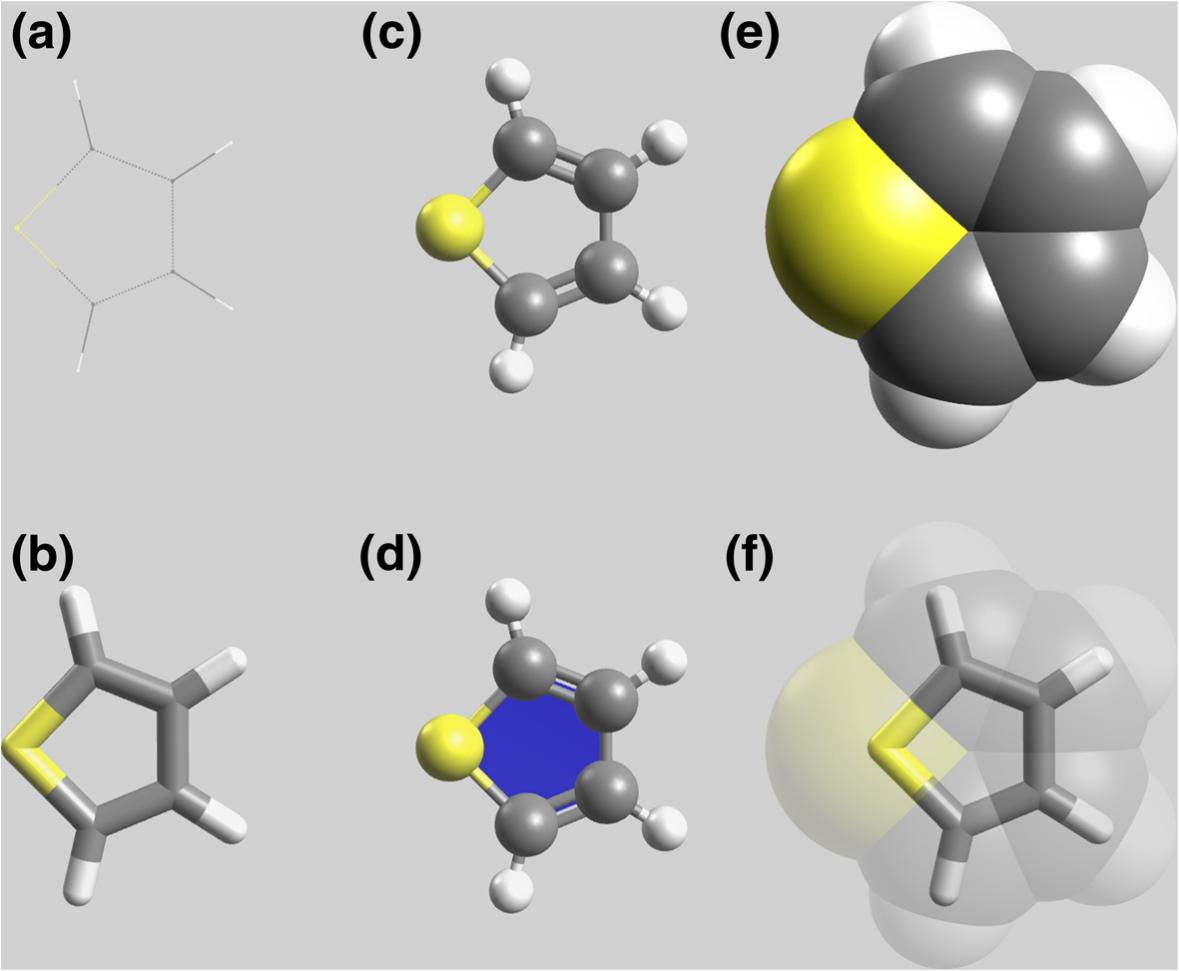
**Standard molecular structure representations.** Several molecular representations of thiophene, (**a**) wireframe, (**b**) stick/licorice, (**c**) ball and stick, (**d**) ball and stick with ring, (**e**) Van der Waals/CPK and (**f**) transparent Van der Waal’s with stick.

Engines are performance critical as the render functions are called each time a frame is requested for display. Efficient rendering is also critical since multiple display types can be combined to form a composite display. For example, ball and stick display overlaid with a transparent Van der Waals space-filling display and ring rendering to highlight all rings in the structure. Figure 2 (d) and (f) show two such combinations of multiple display types.

### Tools

The tools are responsible for virtually all mouse and keyboard interaction with the molecule. A list of all tools is given in Table 2.

**Table 2:** List of default mouse tool plugins

**Name**	**Description**
Draw Tool	Build and edit atoms
Navigate Tool	Move the camera, rotate, pan, and zoom
Bond Centric	Alter bond lengths, angles, and torsions
Manipulate Tool	Move atoms and selected fragments
Select Tool	Select individual atoms, bonds, or fragments
Auto Rotate Tool	Continuously rotate a molecule for presentations
Auto Optimize Tool	Continuously optimize molecular geometry using
	molecular mechanics
Measure Tool	Determine bond lengths, angles, and dihedrals
Align Tool	Rotate and translate to a specified frame of
	reference

The navigation tool provides basic scene navigation, implementing rotation,panning, tilting, and zooming support. The initial point of interaction (where the click occurs) changes the anchor point for navigation; navigation takes place about the center of molecule when clicking in empty space or about the center of any clicked atom. During interaction, the navigation tool provides visual cues to show what type of navigation is taking place. The navigation tool is also used as the default tool if the currently active tool does not handle the mouse event passed to it.

One of the other central tools is the draw tool, which implements a free-hand molecule drawing input method supporting keyboard shortcuts, combo boxes, and a periodic table view to select elements. The user can use the left mouse button to add new atoms or bonds, or click on the bonds to change their order. The right mouse button can be used to delete atoms or bonds, and the directional keys can be used in combination with the mouse to quickly rotate/pan the molecule.

There are also two tools for adjustment of structures (atom or bond centric), a selection tool supporting standard selection interactions, and an auto-rotate tool that allows users to set the speed and angles about which to rotate the molecule. The interactive auto-optimization tool provides a sculpting interaction, where the user can begin a continuous geometry optimization and switch back to the draw or adjustment tools and change the shape and structure of the molecule while observing the new structure being optimized. This can also be combined with the measurement tool to interactively observe bond lengths and angles evolve as the structure is updated and the geometry minimized. If the optimization tool is turned off, the measurement tool also allows the user to precisely adjust bond lengths and/or angles using the adjustment tools.

### Extensions

Extensions represent quite a diverse range of plugins including input generation dialogs for various quantum chemistry codes such as GAMESS, Molpro, NWChem, etc., animation of the molecule, and visualization of molecular orbitals and electron density. Network aware extensions allow the user to click on a menu item to fetch by chemical name and search for “tnt” or “propanol” and have structures returned by the NIH CACTUS Chemical Structure Resolver service [54]. A summary of the extensions distributed with Avogadro is shown in Table 3.

**Table 3:** List of default extension commands

**Name**	**Description**
Create Surfaces	Create surface meshes from molecular orbital/
	electron density data
GAMESS	Prepare input files for GAMESS-US, featuring
	syntax highlighting, advanced properties
Insert Fragment	Insert molecular fragments from a library of
	common fragments
Insert Peptide	Build up and insert peptide fragments
Molecular Mechanics	Use Open Babel’s force fields for geometry
	optimization and conformer searches
MOPAC	Prepare input for and run MOPAC200x
POV-Ray	Ray-trace the displayed structure using POV-Ray
**Properties**	
Angle Properties	Table of all bond angles (editable)
Atom Properties	Table of all atoms with common properties
Bond Properties	Table of all bonds with common properties
Molecule Properties	Common properties of the molecule (including
	molecular weight, etc.)
Torsion Properties	Table of all dihedral angles (editable)
Spectra	Visualize spectra from output files
Super Cell Builder	Expand atoms with space group, replicate
	specified repeats and perform simple bonding
Unit Cell	Change crystallographic unit cell display and
	parameters
Vibrations	Show and animate molecular vibrations

Other extensions translate the entire scene to POV-Ray input, and call POV-Ray to render the molecule using ray tracing techniques to provide higher quality renderings for publication. Various molecular property dialogs are also implemented as plugins, drawing largely on Open Babel functionality to provide an overview of the molecule. Cartesian editors, addition and removal of hydrogens, fragment, SMILES, and peptide insertion are all implemented as extensions showing up in Avogadro menus. More recently a crystallography extension was added, giving access to a much wider range to functionality useful to practitioners in that area, including Miller Plane visualization, slab and surface generation. New builders for nanotubes, nanoparticles, and DNA are also planned for upcoming releases.

### Colors

The color plugins primarily take either double precision numbers or integer values and return an RGB value. The plugins range from the standard color plugin that takes atomic number and returns the standard RGB value for that element through to mapping things like partial change and index to more easily view various aspects of the molecule’s structure.

By defining a plugin interface for coloring atoms, bonds, or residues, developers can easily offer flexible rendering options to highlight important information without requiring a user to tediously set colors on specific atoms or functional groups. Default color plugins are listed in Table 4, illustrating the variety of options. Each plugin is usually only 40-50 lines of C++ code.

**Table 4:** List of default color plugins

**Name**	**Description**
Atom Index Color	Color based on atom ID (from atom 1, 2, etc.)
Charge Color	Color based on predicted electrostatic partial charge
Custom Color	Color all atoms a specific, custom color
Distance Color	Color based on distance from one end of the
	molecule
Element Color	Standard color scheme, giving each atom a color
**(Default)**	defined by its element
Residue Color	Color based on amino acid or nucleic acid residue
	(i.e., glycine, histidine, etc.)
SMARTS Color	Color atoms matching a specific SMARTS pattern
	with a custom color

### Python scripting

Python bindings are provided for all of the core API. Python code can be used in two ways: the first is the interactive Python terminal, and the second is to write Python plugins; extensions, tools, or display types. Writing a Python plugin requires the same functionality to be implemented as a native C++ plugin [55]. The advantage of Python plugins is that it’s easier to make prototypes since no compilation is required. Python plugins can also easily be shared with other users.

The Python bindings interface with the PyQt python bindings for the Qt toolkit, which enables Python code to use all of Qt’s features when writing a plugin. For example, a short Python script can present a window using Qt and render molecules using Avogadro [56-58].

**Figure 3:**
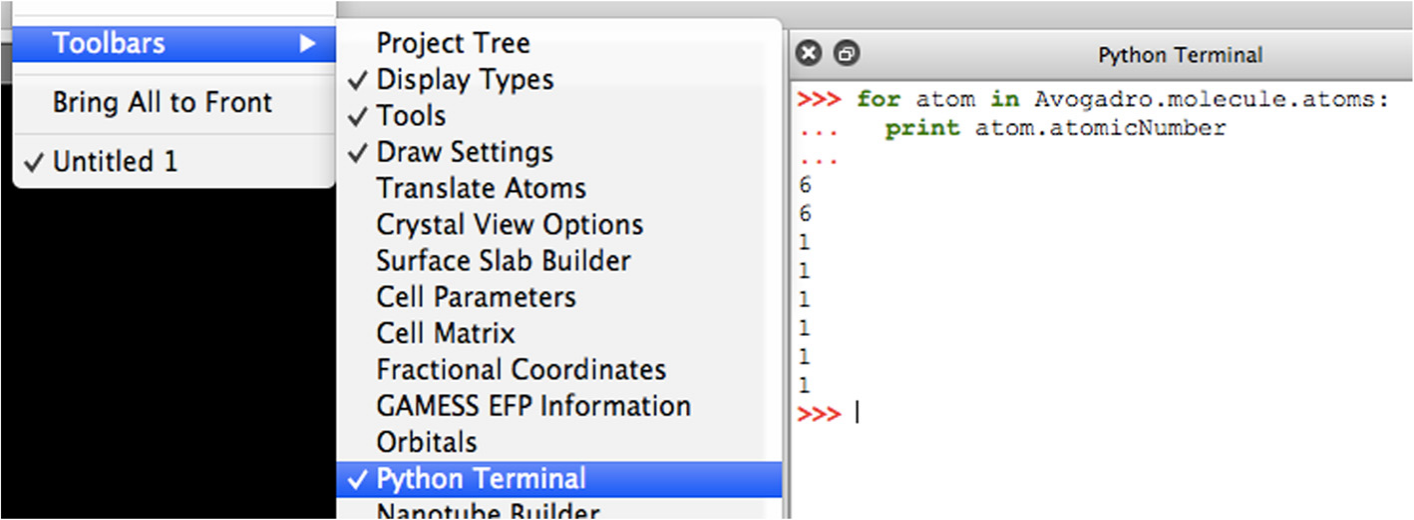
Python scripting terminal, printing atomic numbers.

Avogadro also includes an interactive Python console (Figure 3, which allows users to directly script and manipulate the Avogadro environment [59].

## Results and discussion

### The graphical user interface

**Figure 4:**
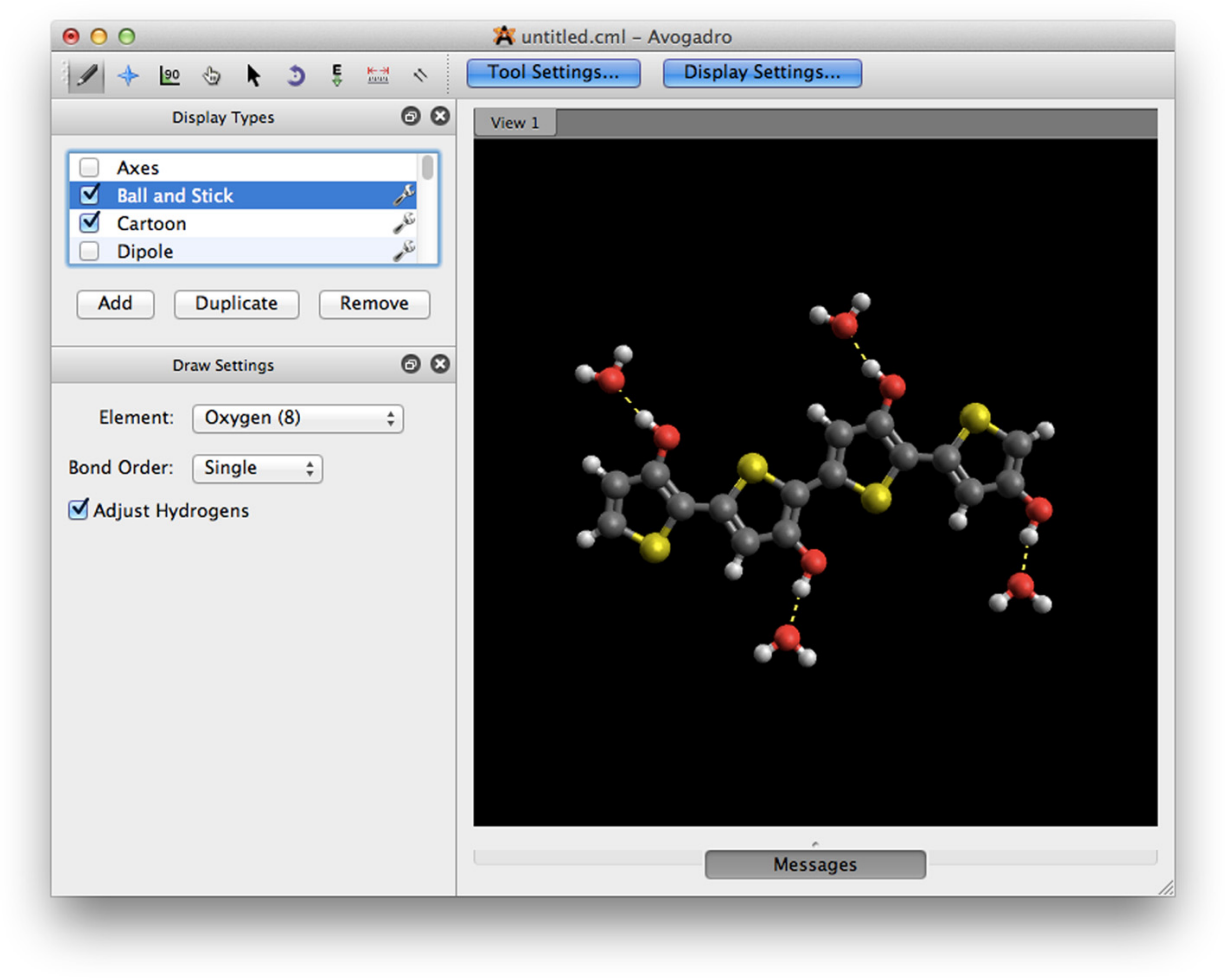
**The Avogadro graphical user interface.** Taken on Mac OS X, showing the editing interface for a molecule.

The first thing most people will see is the main Avogadro application window, as shown in Figure 4. Binary installers are provided for Apple Mac OS X and Microsoft Windows, along with packages for all of the major Linux distributions. This means that Avogadro can be installed quite easily on most operating systems. Easy to follow instructions on how to compile the latest source code are also provided on the main Avogadro web site [60,61] for the more adventurous, or those using an operating system that is not yet supported.

The Qt toolkit gives Avogadro a native look and feel on the three major supported operating systems—Linux, Apple Mac OS X, and Microsoft Windows. The basic functionality expected in a molecular builder and viewer has been implemented, along with several less common features. It is very easy for new users to install Avogadro and build their first molecules within minutes. Thanks to the Open Babel library [51], Avogadro supports a large portion of the chemical file formats that are in common use. The vast majority of this functionality has been written using the interface made available to plugin writers, and is loaded at runtime. We will discuss these plugin interfaces and descriptions of the plugin types later.

### Semantic chemistry

Avogadro has used CML [19,20] as its default file format from a very early stage; this was chosen over other file formats because of the extensible, semantic structure provided by CML, and the support available in Open Babel [51]. The CML format offers a number of advantages over others in common use, including the ability to extend the format. This allows Avogadro and other programs to be future-proof, adding new information and features necessary for an advanced semantically-aware editor at a later time, while still remaining readable in older versions of Avogadro.

Through the use of Open Babel [51], a large array of file formats can be interpreted. When extending Avogadro to read in larger amounts of the output from quantum codes, it was necessary to devote significant development resources to understanding and adding semantic meaning to the quantum code output. This work was developed in a plugin, which was later split out into a small independent library called OpenQube [62,63]. More recently a large amount of work has been done by the Quixote project [64], JUMBO-Converters, and the Semantic Physical Science workshop to augment quantum codes to output more of this data directly from the code. Since CML can be extended, it is possible to reuse existing conventions for molecular structure data, and add new conventions for the additional quantum data.

### Building a molecule: atom by atom

After opening Avogadro a window such as that shown in Figure 4 is presented. By default, the draw tool is selected. Simply left-clicking on the black part of the display allows the user to draw a carbon atom. If the user pushes the left mouse button down and drags, a bonded carbon atom is drawn between the start point and the final position where the mouse is released.

A large amount of effort has been expended to create an intuitive tool for drawing small molecules. Common chemical elements can be selected from a drop down list, or a periodic table can be displayed to select less common elements. Clicking on an existing atom changes it to the currently selected element, dragging changes the atom back to its previous element and draws a new atom bonded to the original. If the bonds are left-clicked then the bond order cycles between single, double, and triple. Shortcut keys are also available, e.g., typing the atomic symbol (e.g., “C-o” for cobalt) changes the selected element, or typing the numbers “1,” “2,” and “3” changes the bond order.

Right clicking on atoms or bonds deletes them. If the “Adjust Hydrogens” box is checked, the number of hydrogens bonded to each atom is automatically adjusted to satisfy valency. Alternatively, this can also be done at the end of an editing session by using the “Add hydrogens” extension in the build menu.

In addition to the draw tool, there are two tools for adjusting the position of atoms in existing molecules. The “atom centric manipulate” tool can be used to move an atom or a group of selected atoms. The “bond centric manipulate” tool can be used to select a bond, and then adjust all atoms positions relative to the selected bond in various ways (e.g., altering the bond length, bond angles, or dihedral angles). These three tools allow for a great deal of flexibility in building small molecules interactively on screen.

Once the molecular structure is complete, the force field extension can be used to perform a geometry optimization. By clicking on “Extensions” and “Optimize Geometry” a fast geometry optimization is performed on the molecule. The force field and calculation parameters can be adjusted, but the defaults are adequate for most molecules. This workflow is typical when building up a small molecular structures for use as input to quantum calculations, or publication quality figures.

An alternative is to combine the “Auto Optimization” tool with the drawing tool. This presents a unique way of sculpting the molecule while the geometry is constantly minimized in the background. The geometry optimization is animated, and the effect of changing bond orders, adding new groups, or removing groups can be observed interactively.

Several dialogs are implemented to provide information on molecule properties and to precisely change parameters, such as the cartesian coordinates of the atoms in the molecule.

### Building a molecule: from fragments

**Figure 5:**
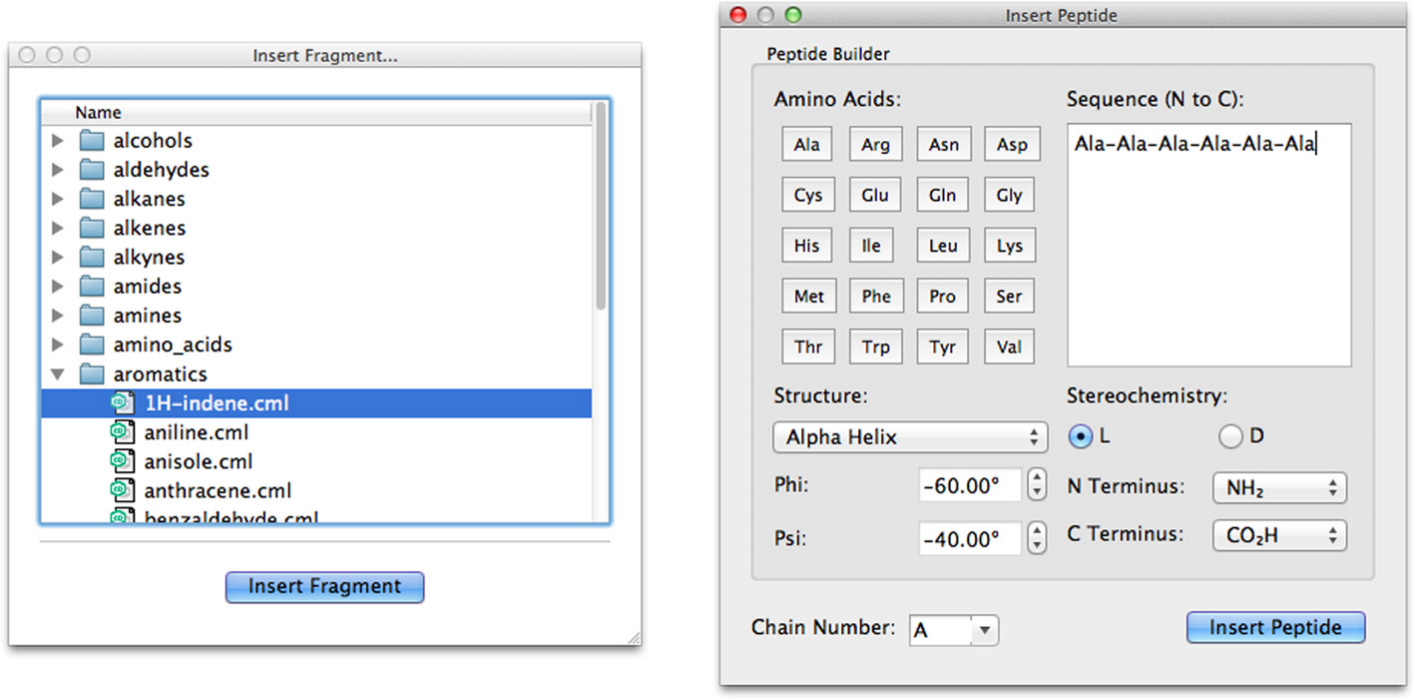
**Dialogs for inserting pre-built fragments.** The left shows molecules, and the right amino-acid sequences.

In addition to building molecules atom-by-atom, users can insert pre-built fragments of common molecules, ligands, or amino-acid sequences, as shown in Figure 5. In all cases, after inserting the fragment, the atom-centered manipulate tool is selected, allowing the fragment to be moved or rotated into position easily.

Users can also insert a SMILES [65,66] string for a molecule. In this case, a rough 3D geometry is generated using Open Babel and a quick force field optimization.

### Preparing input for quantum codes

**Figure 6:**
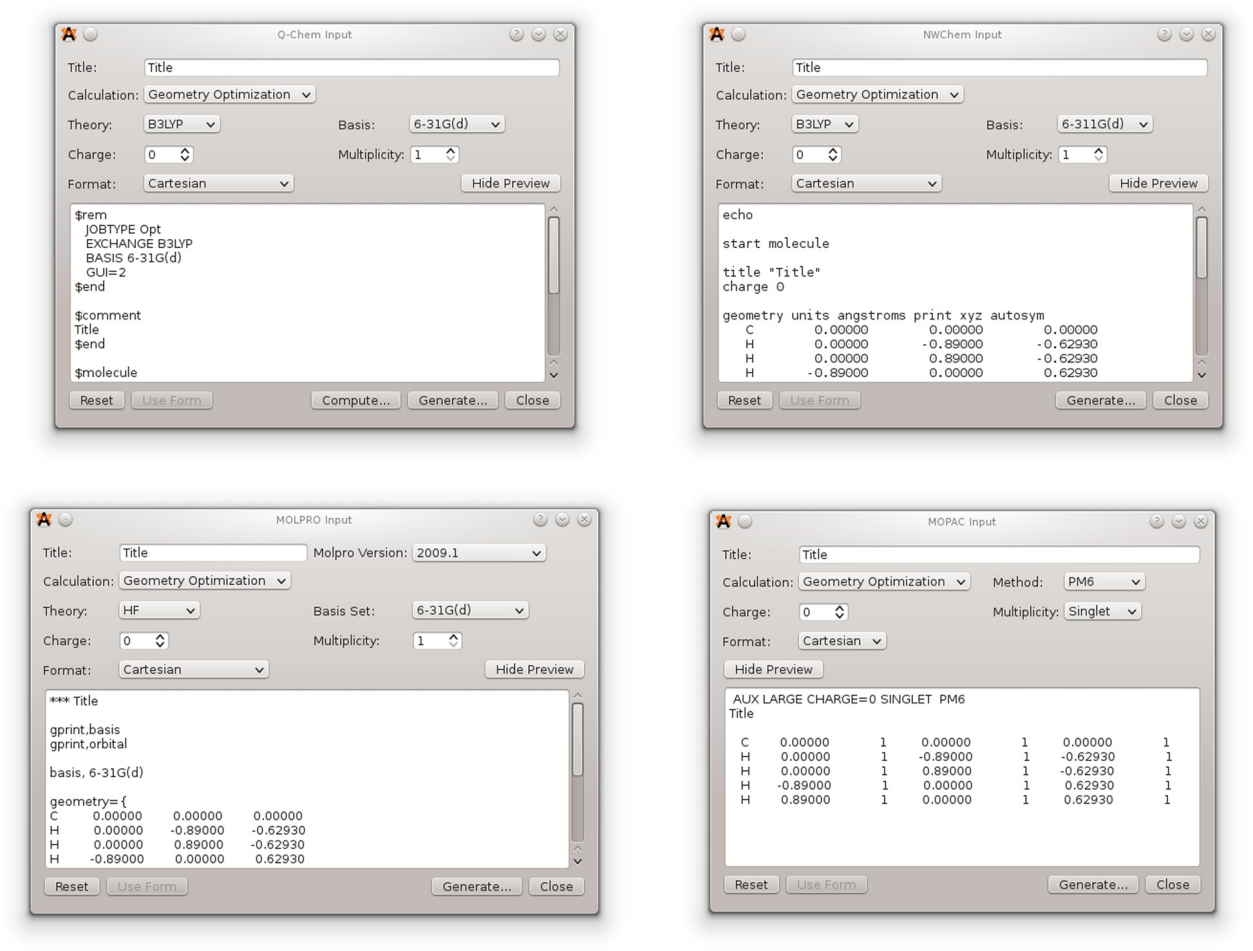
**Dialog for generating input for quantum codes.** Dialogs for generating input for Q-Chem, NWChem, Molpro and MOPAC200x. Note that the dialogs are similar in interface, allowing users to use multiple computational chemistry packages.

Several extensions were developed for Avogadro that assist the user in preparing input files for popular quantum codes such as GAMESS-US, [67] NWChem, [68] Gaussian, [69] Q-Chem, [70] Molpro, [71] and MOPAC200x [72]. The graphical dialogs present the features required to run basic quantum calculations; some examples are shown in Figure 6.

The preview of the input file at the bottom of each dialog is updated as options are changed. This approach helps new users of quantum codes to learn the syntax of input files for different codes, and to quickly generate useful input files as they learn. The input can also be edited by hand in the dialog before the file is saved and submitted to the quantum code. The MOPAC extension can also run the MOPAC200x program directly if it is available on the user’s computer, and then reload the output file into Avogadro once the calculation is complete. This feature will be extended to other quantum codes in future versions of Avogadro.

**Figure 7:**
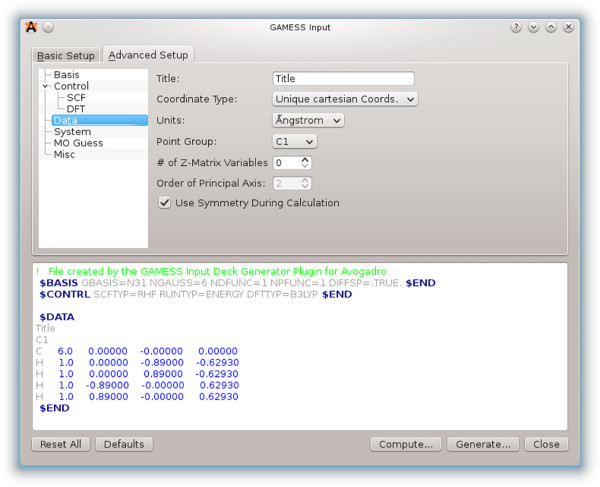
**The GAMESS-US input deck generator.** This input generator has an advanced panel and syntax highlighting.

The GAMESS-US plugin is one of the most highly developed, featuring a basic dialog present in most of the other input deck generators, as well as an advanced dialog exposing many of the more unusual and complex calculation types. In addition to the advanced dialog, the input deck can be edited inline and features syntax highlighting (Figure 7) as used in many popular editors aimed at software developers. This can indicate simple typing errors in keywords, as well as harder to spot whitespace errors that would otherwise cause the hand-edited input deck to fail when being read by GAMESS-US.

### Alignment and measurements

**Figure 8:**
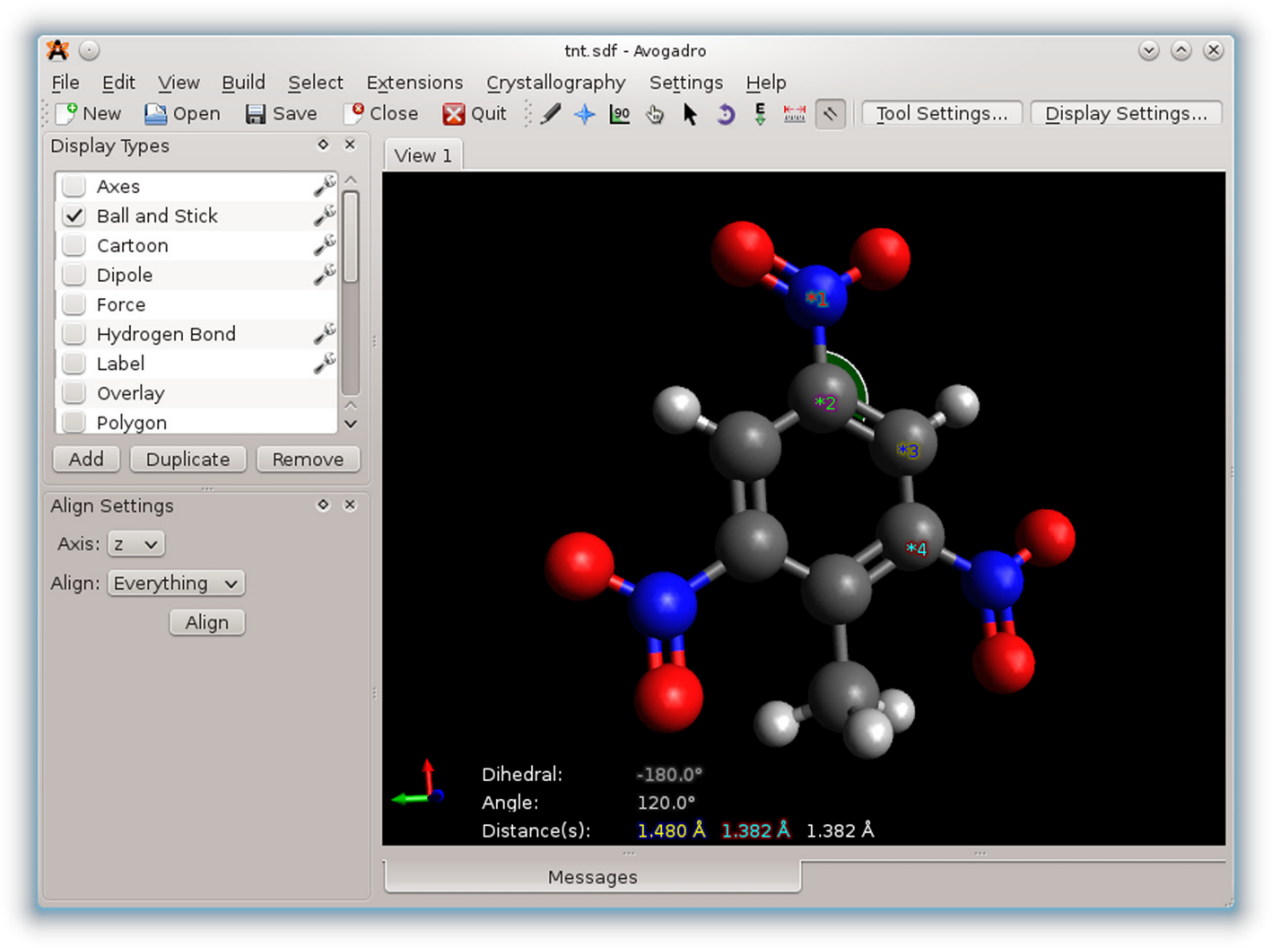
**The measurement tool.** The measurement tool being used to measure bond angles and lengths (on Linux with KDE 4).

One of the specialized tools included in the standard Avogadro distribution is the alignment tool. This mouse tool facilitates the alignment of a molecular structure with the coordinate origin if one atom is selected, and along the specified axis if two atoms are selected. The alignment tool can be combined with the measure, select, and manipulate tools to create inputs for quantum codes where the position and orientation of the molecule is important. One example of this is calculations where an external electric field is applied to the molecule. In these types of calculations, the alignment of the molecule can have a large effect. Figure 8 shows the measurement tool in action with the alignment tool configuration dialog visible in the lower-left corner.

More complex alignment tools for specific tasks could be created. The alignment tool was created in just a few hours for a specific research project. This is a prime example where extensibility was very important for performing research using a graphical computational chemistry tool. It would not be worth the investment to create a new application just to align molecular structures to an axis, but creating a plugin for an extensible project is not unreasonable.

### Visualization

The Avogadro application uses OpenGL to render molecular representations to the screen interactively. OpenGL offers a high-level, cross-platform API for rendering three-dimensional images using hardware accelerated graphics. OpenGL 1.1 and below is used in most of the rendering code, and so Avogadro can be used even on older computer systems, or those without more modern accelerated graphics. It is capable of taking advantage of some of the newer features available in OpenGL 2.0 as described below, but this has been kept as an optional extra feature when working on novel visualizations of molecular structure.

#### Standard representations

In chemistry, there are several standard representations of molecular structure, originally based upon those possible with physical models. The Avogadro application implements each of these representations shown in Figure 2 as a plugin. These range from the simple wireframe representation, stick/licorice, ball and stick, and Van der Waals spheres.

It is also possible to combine several representations, such as ball and stick with ring rendering (Figure 2 (d)), and a semi-transparent Van der Waals space-filling representation with a stick representation to elucidate molecular backbone (Figure 2 (f)).

#### Quantum calculations and electronic structure

Quantum codes were originally developed for line printers, and unfortunately little has changed since then in the standard log files. There are several formats developed for use in other codes and specifically for visualization and analysis, but there is little agreement on any standard file format in the computational quantum chemistry community. A plugin was developed in Avogadro to visualize the output of various quantum codes, and get the data into the right format for further visualization and analysis.

Initially support was added and extended in Open Babel for Gaussian cube files. This format provides atomic coordinates and one or more regularly spaced grids of scalar values. This can be read in, and techniques such as the marching cubes algorithm can be used to compute triangular meshes of isosurfaces at values of electron density for example. Once the code has been developed to visualize these isosurfaces, it became clear that it would be useful to be able to calculate these cubes on the fly, and at different levels of detail depending upon the intended use.

The first format, which was somewhat documented at the time it was developed, is the Gaussian formatted checkpoint format. This format is much easier to parse than the log files generated as the program runs, and provides all of the detail needed to calculate scalar values of the molecular orbital or electron density at any point in space. Once a class structure had been developed for Gaussian type orbitals, the approach was extended to read in several other popular output file formats including Q-Chem, GAMESS-US, NWChem, and Molpro. MOPAC200x support was added later, along with support for the AUX format and Slater type orbitals used in that code. All of these codes output their final configurations using the standard linear combination of atomic orbitals, meaning that parallelization is extremely simple.

**Figure 9:**
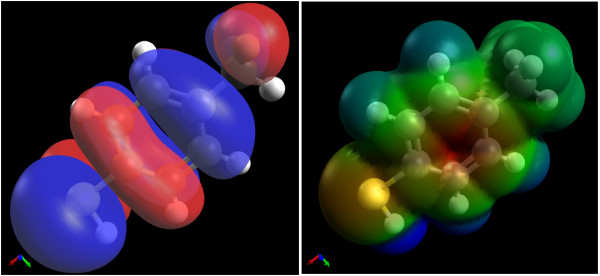
**Molecular orbitals and surfaces.** Rendering of a molecular orbital isosurface (left) and an electrostatic surface potential mapped onto the electron density (right).

The plugin was developed to take advantage of the map-reduce approach offered by QtConcurrent in order to use all available processor cores. This offers almost linear scaling as each point in the grid can be calculated independently of all other points, the results of which can be seen in Figure 9. An alternate approach to calculating the molecular orbitals was developed in a second plugin that has since been split off into a separate project named “OpenQube”. The “OpenQube” library has also been added as an optional backend in VTK during the 2011 Google Summer of Code, bringing support for several output file formats and calculation of cube files that can later be fed into more advanced data pipelines.

A class hierarchy with a standard API is provided for quantum output. Adding support for new codes involved developing a new parser and ensuring the Gaussian or Slater set is populated with the correct ordering and the expected normalization scheme. The s, p, and d-type Gaussian orbitals are supported, with f and g support planned in order to support the increasing number of calculations using these higher-order orbitals. The Basis Set Exchange hosted by EMSL provides access to the basis sets in common use, although at present these basis sets are normally read in directly from the output files. There are several related projects for adding semantic meaning to this type of output, including the JUMBO-Converters project and Quixote. It is hoped that more codes will adopt semantic output in the future, using a common format so that data exchange, validation, and analysis become easier across several codes. This was the subject of a recent meeting with several computational chemistry codes beginning to use FoX in order to output CML. Development has begun on code to read in CML output, either directly from the codes or from conversion of other formats using Open Babel or the JUMBO-Converters. If enough semantic structure can be added to CML, and the converters support a large enough range of the output, this could replace most of the parsing code present in OpenQube. Semantic meaning is one of the most difficult to extract from log files, and coming together as a community will help projects like Avogadro to derive more meaning from the outputs of these codes.

#### Secondary biological structure

Avogadro uses the PDB reader from Open Babel to read in the secondary biological structure. Two plugins exist to process and render this information. The first is a plugin which renders a simple tube between the biomolecule backbone atoms. A second more advanced plugin calculates meshes for the alpha helices and beta sheets. While the first plugin is much faster, the advanced plugin more accurately produces output expected in the field. This allows users flexibility for rendering secondary biological structures.

#### GLSL, novel visualization

GLSL, or OpenGL Shader Language, is a C-like syntax that can be used to develop code that will run on graphics cards and included in the OpenGL 2.0 specification. It has been used to great effect by the games industry, as well as in many areas of data visualization. Several recent papers highlight the potential in chemistry, such as QuteMol [5] in adding support for features such as ambient occlusion to add depth to images.

**Figure 10:**
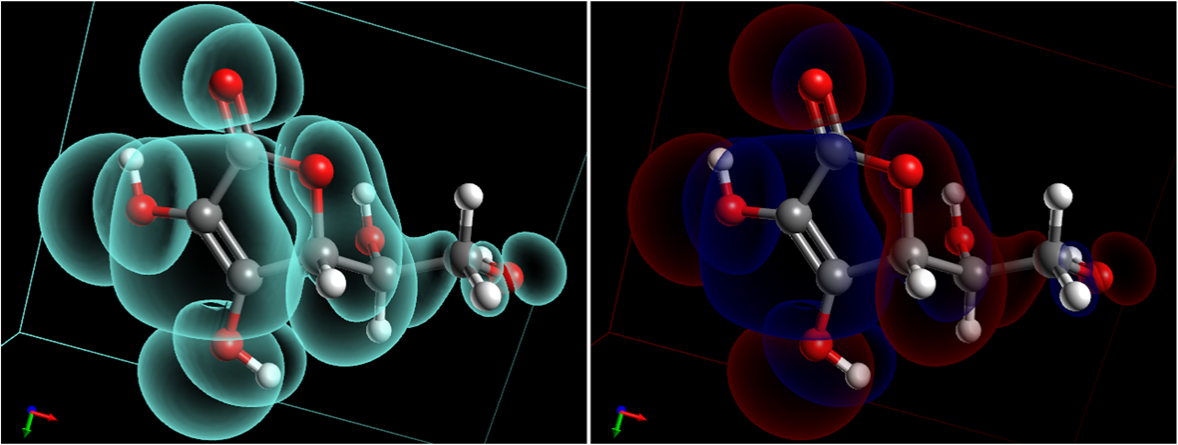
**Molecular orbitals rendering using GLSL shaders.** Rendering of a molecular orbital isosurface using two GLSL shaders to highlight the edges of the surfaces. The X-ray effect (left) and red and blue (right) showing the positive and negative molecular orbital shapes.

Avogadro has support for vertex and fragment shader programs, and several examples are bundled with the package. If the user’s graphics card is capable, these programs can be loaded at runtime and used to great effect to visualize structure. Some of these include summarization techniques such as isosurface rendering where only the edges orthogonal to the view plane are visible, giving a much better rendering of both the molecular and electronic structure (Figure 10).

#### Ray tracing

**Figure 11:**
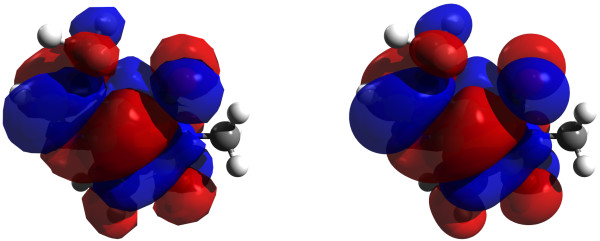
**Ray-traced HOMO isosurfaces of varying cube density.** Rendering of a molecular orbital isosurface using POV-Ray with cubes of low (left) and high (right) density.

Avogadro uses a painter abstraction that makes it much easier for developers to add new display types. It also abstracts away the renderer, making it possible to add support for alternative backends. Currently only OpenGL and POV-Ray are supported. Due to the abstraction, we are able to use the implicit surfaces available in ray tracers to render molecular structure at very high levels of clarity and with none of the triangle artifacts present in standard OpenGL rendered images. Much higher quality transparency and reflection also allow for the images to be used in poster and oral presentations as well as research articles (Figure 11).

This feature is implemented in an extension, with an additional painter class deriving from the base class and a dialog allowing the user to edit the basic rendering controls. The POV-Ray input file can also be retained and edited to produce more complex images, or to allow for much finer control of the rendering process if desired.

### Avogadro library in use

**Figure 12:**
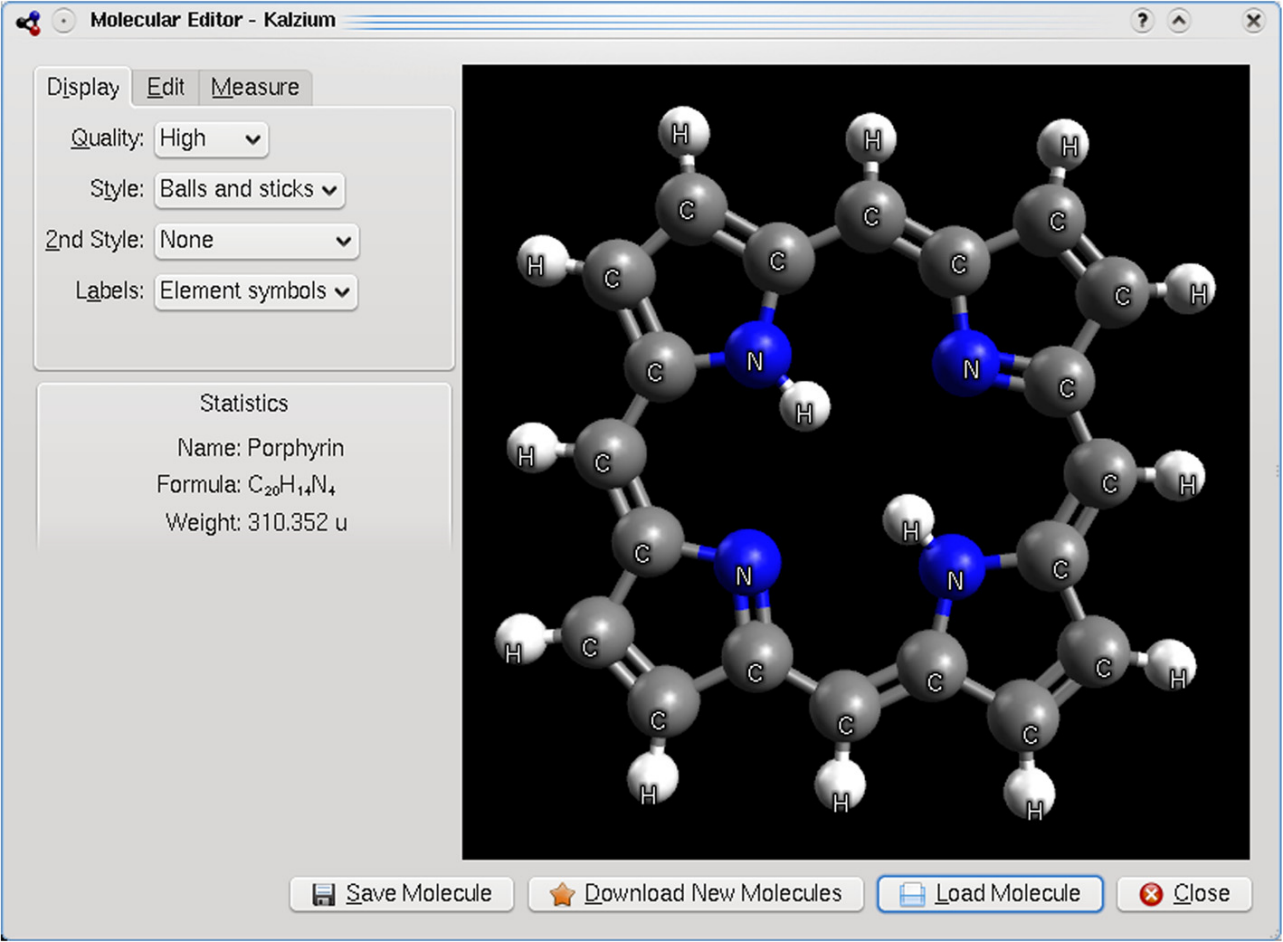
The Kalzium application in KDE using Avogadro to render molecular structures.

The Avogadro library’s first use was the Avogadro application, closely followed by the Kalzium periodic table program that is part of the KDE software collection. This initial work was funded in part by the Google Summer of Code program in 2007, and also resulted in the addition of several other features in the Avogadro library to support Kalzium and general visualization and editing of molecular structure (Figure 12).

The Q-Chem package [70] has developed “QUI - The Q-Chem User Interface” [73] around Avogadro, originally as an Avogadro extension. This is a more advanced version of the input generator developed in Avogadro, with much tighter integration. Molpro [71] has also published some results from their development of a Molpro interface using the Avogadro library [74].

#### Packmol

**Figure 13:**
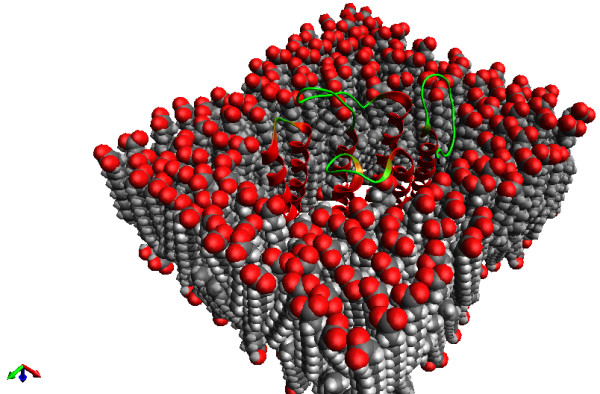
The PackMol lipid layer as produced by the PackMol extension.

Packmol is a third-party package designed to create initial “packed” configurations of molecules for molecular dynamics or other simulations [75,76]. Examples include surrounding a protein with solvent, solvent mixtures, lipid bilayers, spherical micelles, placing counterions, adding ligands to nanoparticles, etc. Typically, users may have equilibrated “solvent boxes” which have been run for long simulations to ensure proper density, and both short and long-range interactions between solvent molecules. Using such solvent boxes allows placing solute molecules, such as proteins, in an approximately correct initial structure, such as that shown in Figure 13. The solute is added into the box, and solvent molecules with overlapping atoms are removed. While these utilities are often enough, creating complex input files is not always easy. For more complicated systems, Packmol can create an initial configuration based on defined densities, geometries (e.g., sphere, box, etc.), and the molecules to be placed. An Avogadro developer wrote an external plugin to facilitate use of Packmol, including estimating the number of molecules in a given volume.

The plugin is not currently distributed with Avogadro as a standard feature, although it is planned for some future version. It serves as an example of how Avogadro can facilitate a workflow with a text-oriented package (Packmol), including saving files in the PDB format required by Packmol, generating an input file, and reading the output for visualization, analysis, and further simulations.

#### XtalOpt

**Figure 14:**
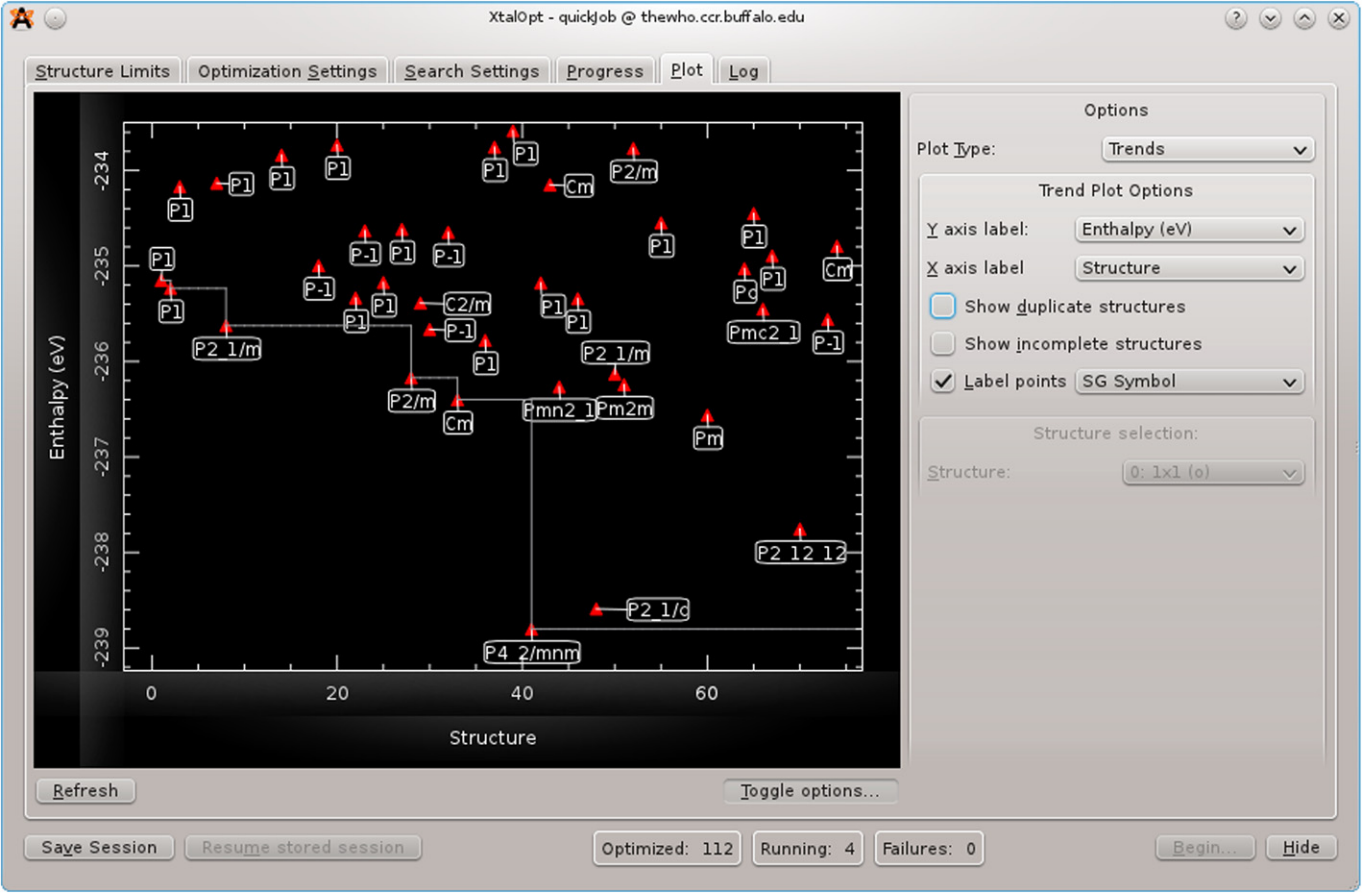
**The XtalOpt extension.** XtalOpt extension showing a plot of stability vs. search progress for a TiO_2_ supercell.

The XtalOpt [77,78] software package is implemented as a third-party C++ extension to Avogadro and makes heavy use of the libavogadro API. The extension implements an evolutionary algorithm tailored for crystal structure prediction. The XtalOpt development team chose Avogadro as a platform because of its open-source license, well-designed API, powerful visualization tools, and intuitive user-interface. XtalOpt exists as a dialog window (Figure 14) and uses the main Avogadro window for visualizing candidate structures as they evolve. The API is well suited for XtalOpt’s needs, providing a simple mechanism to allow the user to view, edit, and export the structures generated during the search. Taking advantage of the cross-platform capabilities of Avogadro and its dependencies, XtalOpt is available for Linux, Windows, and Mac.

## Conclusions

Avogadro has grown over its first six years to become an important tool for building, editing, visualizing, and analyzing chemical and molecular data. With over 270,000 downloads, language translations and localizations, and over 100 citations, it has become an integral part of the chemical software toolbox. Through use of the native CML file format and a wide variety of chemical data import, Avogadro can provide semantic chemical data editing and conversion. We seek to provide an integrated environment in the simulation and cheminformatics workflow. While more must be done, particularly in regards to documentation, tutorials, ease-of-use, and automation, we aim to improve the quality and feature set with each new release.

Currently, two upcoming versions of Avogadro are under development. The first is Avogadro version 1.1, which adds additional features and refinement, particularly including crystallography support developed through the XtalOpt project. The second is a more substantial development for Avogadro version 2.0, where many of the core data structures are being rewritten in order to offer greater flexibility and scalability. Our goal is to support an increasing scope of chemical systems, including biomolecules (DNA, RNA, saccarides, etc.), materials (crystallography, polymers, surfaces), nanoscience (nanoparticles, nanotubes, graphene, etc.) with improved speed, intuitive ease-of-use and simpler non-reciprocal licensing terms.

Avogadro is freely available from http://avogadro.openmolecules.net/, and new contributors are welcome in all areas (users, developers, testers, translators, educators, students, researchers, dreamers).

## Availability and requirements

**Project Name:** Avogadro **Project home page:**http://avogadro.openmolecules.net/**Operating system(s):** Cross-platform **Programming language:** C++, bindings to Python **Other requirements (if compiling):** CMake 2.6+, Open Babel, Qt 4.6+, Eigen 2 **License:** GNU GPL v2 **Any restrictions to use by non-academics:** None additional

## Competing interests

The authors declare that they have no competing interests.

## Authors’ contributions

GRH and DEC are the founders of the Avogadro project. MDH is the current lead developer and maintainer of Avogadro. GRH, DL and TV are active developers. DL and EZ are founders of the XtalOpt project which is discussed in this work. TV developed the PackMol plugin. All authors read and approved the final manuscript.
